# Reinforcement of the bio-gas conversion from pyrolysis of wheat straw by hot caustic pre-extraction

**DOI:** 10.1186/s13068-018-1072-5

**Published:** 2018-03-19

**Authors:** Lilong Zhang, Keli Chen, Liang He, Lincai Peng

**Affiliations:** Faculty of Chemical Engineering, Kunming University of Science and Technology, Kunming, 650500 Yunnan China

**Keywords:** Wheat straw, Soda extraction, Low temperature, Highly selective, Pyrolysis products, Pyrolysis behaviour

## Abstract

**Background:**

Pyrolysis has attracted growing interest as a versatile means to convert biomass into valuable products. Wheat straw has been considered to be a promising biomass resource due to its low price and easy availability. However, most of the products obtained from wheat straw pyrolysis are usually of low quality. Hot soda extraction has the advantage of selective dissolution of lignin whilst retaining the carbohydrates. This can selectively convert biomass into high-quality desired products and suppress the formation of undesirable products. The aim of this study was to investigate the pyrolysis properties of wheat straw under different hot caustic pretreatment conditions.

**Results:**

Compared with the untreated straw, a greater amount of gas was released and fewer residues were retained in the extracted wheat straw, which was caused by an increase in porosity. When the NaOH loading was 14%, the average pore size of the extracted straw increased by 12% and the cumulative pore volume increased by 157% compared with the untreated straw. The extracted straw obtained from the 14% NaOH extraction was clearly selective for pyrolysis products. On one hand, many lignin pyrolysis products disappeared, and only four main lignin-unit-pyrolysis products were retained. On the other hand, polysaccharide pyrolysis products were enriched. Both propanone and furfural have outstanding peak intensities that could account for approximately 30% of the total pyrolysis products. However, with the excessive addition of NaOH (i.e. > 22% w/w) during pretreatment, the conversion of bio-gas products decreased. Thermogravimetric and low-temperature nitrogen-adsorption analysis showed that the pore structure had been seriously destroyed, leading to the closing of the release paths of the bio-gas and thus increasing the re-polymerisation of small bio-gas molecules.

**Conclusions:**

After suitable extraction (14% NaOH loading extraction), a considerable amount (25%) of the soluble components dissolved out of the straw. This resulted in an increase in both pore size and volume. This condition appeared to be optimally selective for the release of value-added pyrolysis products such as furfural, ketones and lignin monomer units. However, excessive addition of alkali (22%) for extraction could change the original interior structure, resulting in a decrease in both pore size and volume. This interior structure modification limited the release of pyrolysis products, and greater carbonisation occurred. 
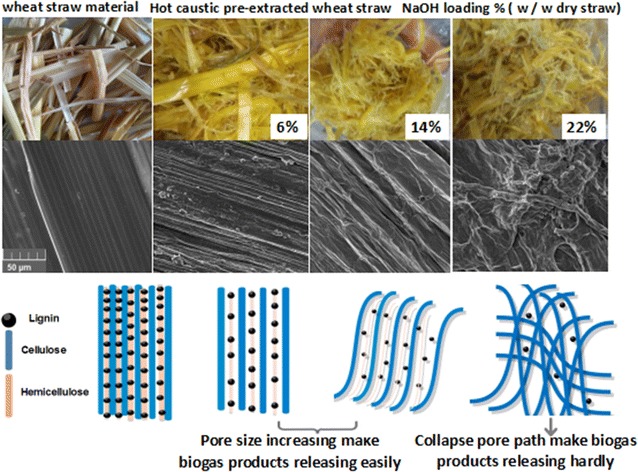

**Electronic supplementary material:**

The online version of this article (10.1186/s13068-018-1072-5) contains supplementary material, which is available to authorized users.

## Background

Wheat straw is the second-most abundant agricultural residue in the world [1]. China could produce a wheat crop of 22 million tons annually, and the yield of wheat straw would then reach 30 million tons [2, 3]. In many developing countries, wheat straw is currently treated mainly by land-filling or direct incineration, which could bring about serious air pollution and a waste of bio-resources. Considering the low economic benefits and stringent regulations for environmental protection, effective recycling of the waste biomass in an economically feasible manner is still a considerable challenge [4]. Therefore, an environmentally friendly and economically feasible method should be developed for the treatment of this biomass waste.

Pyrolysis, as an environmentally friendly and cost-effective technology for recycling of biomass, has several advantages over traditional treatments such as incineration and land-filling because of its low energy consumption. Only approximately 10% of the energy content of biomass is consumed for the pyrolysis itself [5, 6]. At same time, the harmful gas released in the biomass pyrolysis process is remarkably decreased compared with that from incineration [7].

Studies on the conversion of whole-crop cereals into energy and valuable chemicals by the pyrolysis process show very promising future applications. Vispute [8] has found that pyrolysis fuel can be converted into industrial commodity chemical feedstocks using an integrated catalytic approach that combines hydro-processing with zeolite catalysis. Given the availability of biomass, pyrolysis oil and gas can be advantageous when compared with the less-reliable supply and fluctuating prices of petrol. Some areas which are rich in biomass resources could provide locally sourced biomass to produce pyrolysis oil and gas [9–11].

Although pyrolysis provides a promising means for the production of various chemicals, most of the products obtained from biomass pyrolysis are usually of low quality. Especially for the straw, the pyrolysis products usually contain low-quality bio-gas, bio-char and low-calorie bio-fuel [12]. The selective conversion of biomass into the desired products of high quality (e.g. bio-oil and bio-char) and suppression of the formation of undesired products (e.g. gas and tar) remain a difficult challenge.

The biggest obstacle is the complex composition of natural biomass. Cellulose chains are packed by hydrogen bonding in fibrils. These fibrils are attached to each other by hemicelluloses, pectin, and lignin [13]. These strong chains form a crystalline ribbon which is the biggest obstacle to chemical access, and consequently results in low bio-conversion efficiency [14]. Pretreatment is regarded as a process that makes biomass more amenable to hydrolysis than in its original state. It can make the raw material more accessible to reactivity due to the increased availability of contact surfaces and decreased crystalline structure [15]. Soda extraction has been considered as a promising chemical extraction method due to its low operating cost, better infiltration of agricultural stalks, and reduction in holocellulose degradation. The traditional alkali extraction temperature has usually been above 130 °C. At this temperature, over 50% of the mass can be extracted with alkali, which has been regarded as one important method to gain high-value constituents such as hemicellulose from biomass [16]. However, the effects of hot (90 °C) caustic extraction on the pyrolysis behaviour and products of wheat straw have not yet been reported.

Our previous paper [17] presented a study of the soda extraction of wheat straw using a wider range of NaOH dosages under low-temperature conditions (90 °C). We found that soda extraction has the advantage of selective dissolution of lignins, whilst carbohydrates were retained. Because of the low-temperature limitation, the extracted straw forms pulp with difficulty. In addition, the black liquor (BL) derived from the traditional straw pulping process cannot be used directly for alkali recovery due to problems with silica, the lower efficiency of alkali recovery, etc. Based on the discussion above, pyrolysis is a good choice for soda-extracted straw. Whether it can be used for the pyrolysis process depends on an understanding of pyrolysis behaviour.

## Results

### Composition of the extracted straw

Soda extraction of lignocellulosic materials is a process during which the alkaline lignin, carbohydrate, ash, and alkaline undissolved matter are dissolved out. The percentage yields of the components retained in the extracted straw are displayed in Table 1. The loss in mass of the wheat straw after extraction with 6% NaOH was approximately 3–5%. The loss increases to 15–25% at 14% NaOH loading. When the NaOH loading reached 22% and the pretreatment time was prolonged to 3 h, more than 30% of the composition of the straw was dissolved out by the extraction. Cellulose and lignin are the main components of the extracted straw. Before the NaOH loading reached 14%, the content of cellulose in the extracted straw remained basically stable. The percentage yield of cellulose in the extracted straw only slightly decreased (ca. 2%) when the NaOH loading reached 22%.

**Table 1:** Composition of the extracted straw

Percent yield of the components %	Raw material	Pretreatment NaOH loading (w/w dry straw)								
		6%			14%			22%		
		1 h	2 h	3 h	1 h	2 h	3 h	1 h	2 h	3 h
Ash	7.05	6.73	6.31	5.24	5.45	5.22	5.13	4.05	4.39	4.82
Cellulose	41.72	43.35	43.45	44.50	41.08	42.18	43.64	40.89	40.62	40.21
Pentosan	23.15	20.04	18.09	16.11	15.23	13.33	12.74	12.33	11.13	8.21
Klason lignin	21.42	19.70	18.32	15.69	10.00	9.72	8.73	7.92	5.31	4.82
Acid-soluble lignin	2.15	2.06	1.75	1.27	1.38	1.13	1.02	1.14	1.06	0.99
Residual yield	100	97.05	92.86	87.47	76.90	75.50	73.94	67.86	62.85	58.28

### Effect of soda extraction on the average pore size and specific surface area of straw

Table 2 shows the average pore size, cumulative pore volume, and specific surface area of the extracted straw at different NaOH loadings. The average pore size and volume of the extracted straw increased. When the NaOH loading was 14%, the average pore size of the extracted straw increased by 12% and the cumulative pore volume increased by 157% compared with raw material. However, after extraction with an excessive alkali charge of 22%, the collapse of the fibres could bring about the decrease in the pore size and volume. Similar to the tendency of the pores, the BET specific surface area decreased after soda extraction.

**Table 2:** Average pore size and BET surface area of soda-extracted straw at different NaOH loadings

Extracted NaOH loading (w/w dry straw), %	Average pore size (nm)	Cumulative pore volume (cm^3^/g)	BET specific surface area (m^2^/g)
Unextracted	13.90	0.01	0.47
6	15.07	0.02	0.48
14	15.57	0.03	0.67
22	14.43	0.02	0.61

### Elemental analysis of the extracted straw

Organic elements, the main decomposition products, play an important role in pyrolysis behaviour. Table 3 shows the concentration of each organic element in the extracted straw. It can be found that soda extraction is helpful for increasing the C and H content in the extracted straw. Especially for a low NaOH loading or short pretreatment time under high NaOH loading, the extracted straw has a high proportion of C and H in the extracted straw. Clearly, an increase in the content of C and H is good for improving the heating value (HV).

**Table 3:** Elemental analysis of raw material and extracted straw

	Organic elements content, wt/% (h)	N	C	H	S	O^a^
6%	Raw material	0.43	45.18	6.31	0.40	46.67
	1	0.40	48.18	6.90	0.27	44.26
	2	0.36	46.81	6.69	0.07	46.08
14%	3	0.36	46.03	6.71	0.01	46.89
	1	0.22	46.01	6.69	0.01	47.08
	2	0.17	45.58	6.40	0.01	47.83
22%	3	0.11	44.98	6.55	0.01	48.36
	1	0.19	45.39	6.59	0.01	47.82
	2	0.11	44.14	6.58	0.01	49.16
	3	0.12	44.64	6.48	0.01	48.74

^a^Elemental O by difference

In addition to those three dominant elements, straw also contains small amounts of the elements such as nitrogen (N) and sulphur (S). Almost all of the S compounds could be dissolved out after alkali extraction whilst only 55% of the N was removed at an NaOH loading of 14%. Thus, soda extraction does little work on the dissolution of protein during soda extraction at low NaOH loading. However, when the NaOH loading reaches 22%, the micro-structure is exposed at the surface, and protein could be pulled into the lye solution from the straw. Compounds containing N account for only approximately 10% of all the organic elements in the extracted straw.

### Effect of soda extraction on pyrolysis behaviour using TG–DTG

According to the pyrolysis process, the thermogravimetric analysis (TG) and differential thermal analysis (DTG) curves can be divided into three stages. The first stage was from room temperature to 200 °C, the second stage from 200 to 350 °C, and the third stage from 350 to 600 °C. For the first stage, because of the removal of moisture, the sample weight changed only slightly, with almost no drying peaks.

**Fig. 1:**
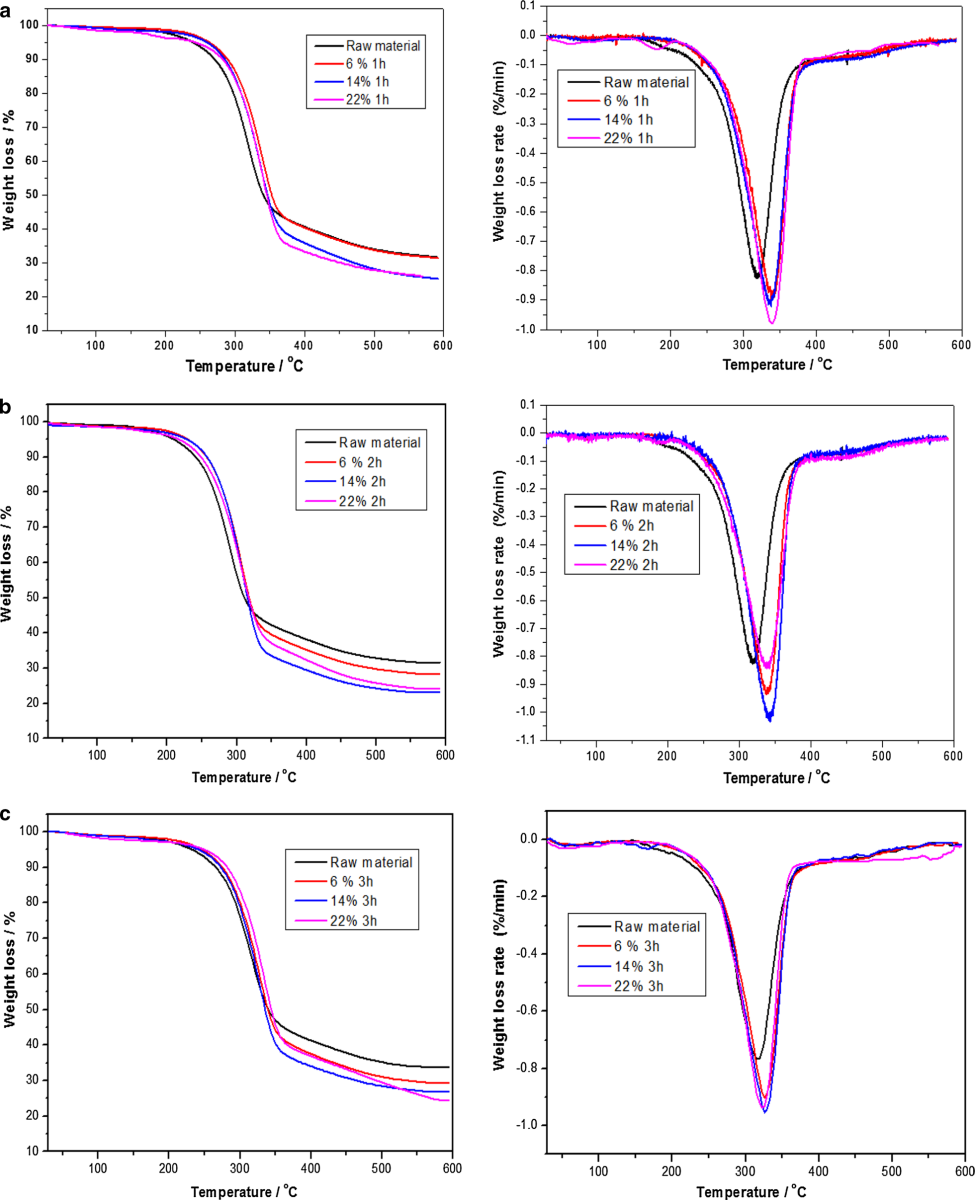
Pyrolysis characteristics of raw material and extracted straw at different pretreatment time **a** 1 h, **b** 2 h, and **c** 3 h

The second stage occurred between 200 and 350 °C, which was the main decomposition stage of hemicellulose, cellulose, and lignin. The weight loss of wheat straw clearly varied under different soda-extraction conditions. As Fig. 1 shows, after alkali extraction with different NaOH loadings, the straw has a similar temperature for the maximum weight loss rate at the same pretreatment time. This indicates that pretreatment time has a greater impact than NaOH loading on the maximum weight loss rate. As is well known, unextracted straw contains greater low-molecular-weight (LMW) mass which could be cracked during a second pyrolysis process. So, a lower pyrolysis temperature could be gained from the use of unextracted straw. The extracted straw has a higher temperature for the maximum weight loss rate than that for unextracted straw. By prolonging the pretreatment time, this temperature gap is diminished from 50 to 15 °C. This gap appeared constantly during whole pyrolysis process.

In Fig. 1b, the maximum weight loss rate of extracted straw increased with an increase in the NaOH loading. The maximum weight loss rate increased from 0.88%/°C at 6% for 1 h to 0.98%/°C at 22% for 1 h. Pretreatment at 2 and 3 h exhibited a different regular pattern. After alkali extraction, the fibre could sufficiently swell after a long pretreatment time. The deformed, bent and even curled fibre is favoured for pyrolysis. The curve at 14% for 2 h has the highest weight loss rate of − 1.05%/min, whilst those curves at 6% for 2 h and 22% for 2 h have peaks at − 0.85 and − 0.95%/min, respectively. This finding may be due to the composition of the pretreated straw. A loading of 14% NaOH for 2 h was the optimal condition for lignin removal whilst minimising the loss of carbohydrate. The pyrolysis temperature for carbohydrate is lower than that of lignin. Thus, a greater carbohydrate content in the pretreated straw could bring about a lower pyrolysis temperature with a higher weight loss rate. This finding indicates that lignin removal could facilitate wheat straw pyrolysis and improve the release of volatile components.

The third stage occurred above 350 °C and the loss in weight amounts to approximately 15% of the entire weight loss. The residues are mainly fixed carbon and a small amount of ash (20%), which is approximately 25–33% of the unextracted straw. It is worth noting that with an increase in the NaOH loading and prolonging of the pretreatment time, the residue content decreased. Thus, straw could produce more pyrolysis products after alkali extraction with a high conversion rate. Of course, not all extracted straw could produce such a favourable effect. As shown in Fig. 1, straw after alkali extraction with 6% NaOH loading has a temperature–weight loss curve similar to that of unextracted straw. When the NaOH loading is increased to 14% or higher, more gas is released, and fewer residues remain. However, straw after extraction using a 22% NaOH loading has a greater amount of remaining residue.

### Effect of soda extraction on pyrolysis products using TG–FTIR analysis

**Fig. 2:**
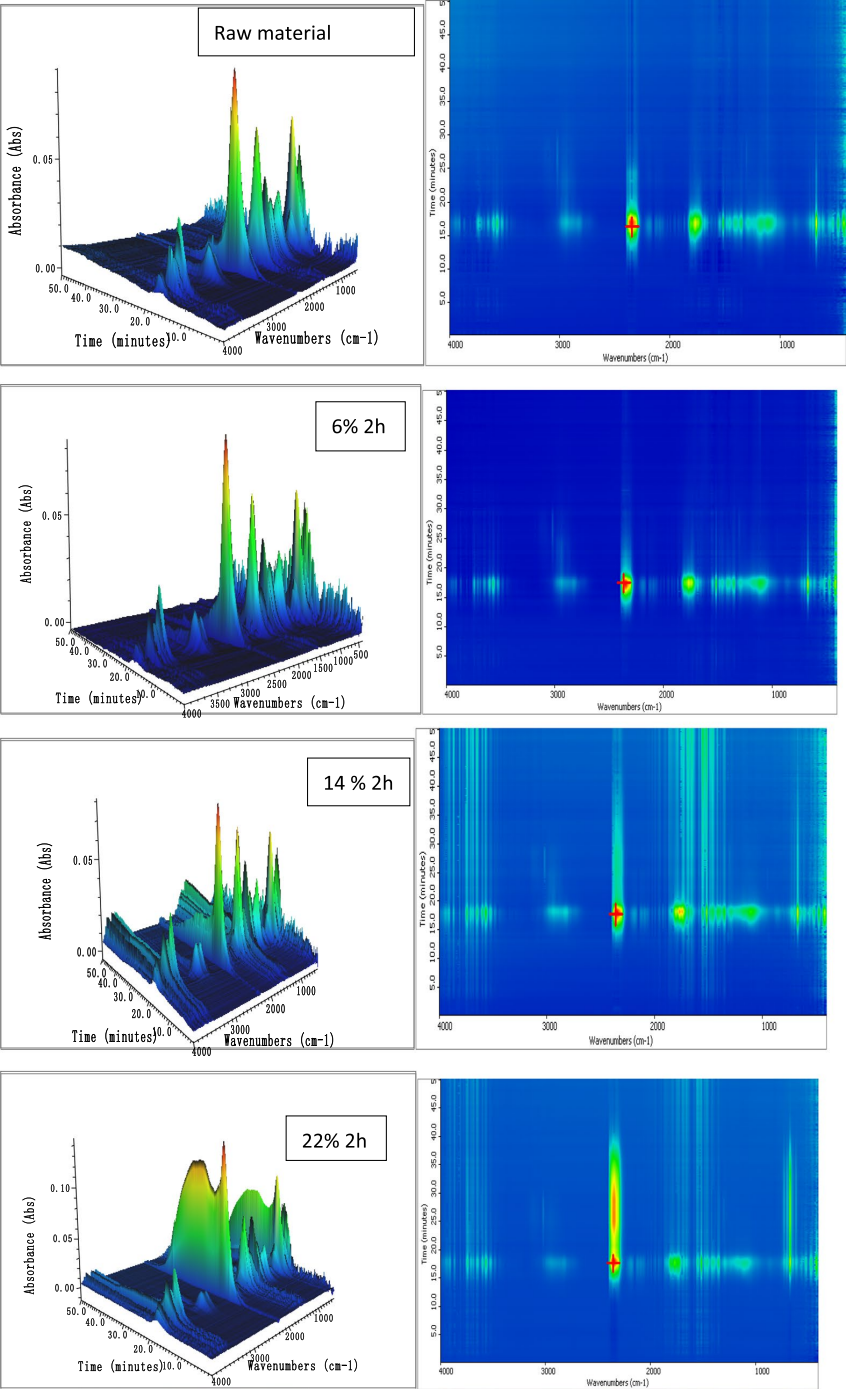
Three-dimensional infrared spectra of gases evolved during pyrolysis

The species of pyrolysis products during the thermal degradation process were monitored and analysed by real-time FTIR (Fourier transform infrared spectroscopy). The typical 3D infrared spectra of the soda-extracted straw for 2 h are representative and their top views are presented in Fig. 2. The corresponding functional groups and the IR signal with the possible compounds are listed in Table 4. Since mutual overlaps exist in many characteristic peaks, gas products could not be completely reflected by an infrared spectrum. Previous work on the pyrolysis products from biomass could help us analyse the composition of the products and the temperatures at which they occur [18].

**Table 4:** The types of main volatiles from different extracted residual pyrolysis

Wave number (cm^−1^)	Functional group	Compounds
3964–3000	O–H	Water
3200–2850	C–H stretch	Alkanes
2860–2770	C=O stretch	Aldehydes
2217–2391	C=O stretch	Carbon dioxide
2150–2122	C=O stretch	Carbon monoxide
1507	Aromatic skeleton vibration	Aromatic
1300–1400	O–H	Phenols
1040–1180	C–H stretch OH or C–O bending stretch	Alcohols
965–1435		Furaldehyde (R)

As Fig. 2 shows, after a low NaOH loading of 6%, the pyrolysis behaviour of extracted straw has 3D FTIR spectra similar to those of unextracted straw. Extracted straw using high alkali loadings (14 and 22%) could release similar pyrolysis products from approximately the same temperatures. In the details of each spectrum, each sample has its special peaks. Overall, the main loss from each of the four samples appeared in a temperature range of 300–500 °C (15–30 min). These gas products could be classified by the degree of pyrolysis: CO_2_ and H_2_O are the completely pyrolysed products, methane and alcohols are cleavage micromolecules, and aromatic compounds and furaldehyde are pyrolysis units.

CO_2_ and H_2_O are the main degradation products of biomass and account for approximately 30% of the mass balance. The release of CO_2_ for raw straw material mainly occurred in the temperature region of 200–400 °C. Compared with the raw material, extracted straws have a larger temperature range in the CO_2_ curve [19]. With an increase in the NaOH loading, the intensity of the CO_2_ peak also increases. When the NaOH loading reaches 22%, the release of CO_2_ has a wider temperature range of 200–800 °C, with the peak bands of maximum absorption intensity at approximately 350 and 650 °C.

There is no release process for H_2_O synchronous with that of CO_2_. The curve for H_2_O reaches its peak at an NaOH loading of 14%, and the absorbance remains relatively stable with an increase in the NaOH loading. It has a release process similar to that of aromatic compounds, which is in the region of 1700–1300/cm. This kind of product could result from the dehydration of carbohydrate and cleavage of hydroxyl groups from alkyl chains on aromatic rings. At same time, the small organic molecules including furans, phenols, ketones, and aldehydes could be formed simultaneously with the release of H_2_O. In this species region, the straw after extraction at a 14% NaOH loading could release volatile organics at a higher intensity compared with the other three samples. Especially for phenol in the range of 1300–1400/cm, extracted straw after alkali extraction at a 14% NaOH loading has an outstanding sharp peak in this region at approximately 350 °C. It means that higher concentrations of integrated pyrolysis unit structures could be gained from alkali-extracted straw using a 14% NaOH loading. In other words, straw extracted at a 14% NaOH loading has the highest selectivity for producing monomer units of lignin and carbohydrate.

### Effect of soda extraction on pyrolysis products using Py–GC/MS analysis

For Py–GC/MS results, the chromatographic percentage of the integrated peak area is linear with its content. Some representative compounds for each category were selected due to their relatively higher percentage of peak integral area. The pyrolysis products are very complex and contain numerous compounds. Here, gas products with concentrations above 5% of the total products were chosen for pyrolysis analysis. Some components were analysed, but they were not present because of their low concentrations. Although it was not possible to analyse every product, the pyrolysis products can be divided into two main categories according to the functional groups detected by Py–GC/MS: polysaccharide-derived products and lignin-derived pyrolysis products.

**Fig. 3:**
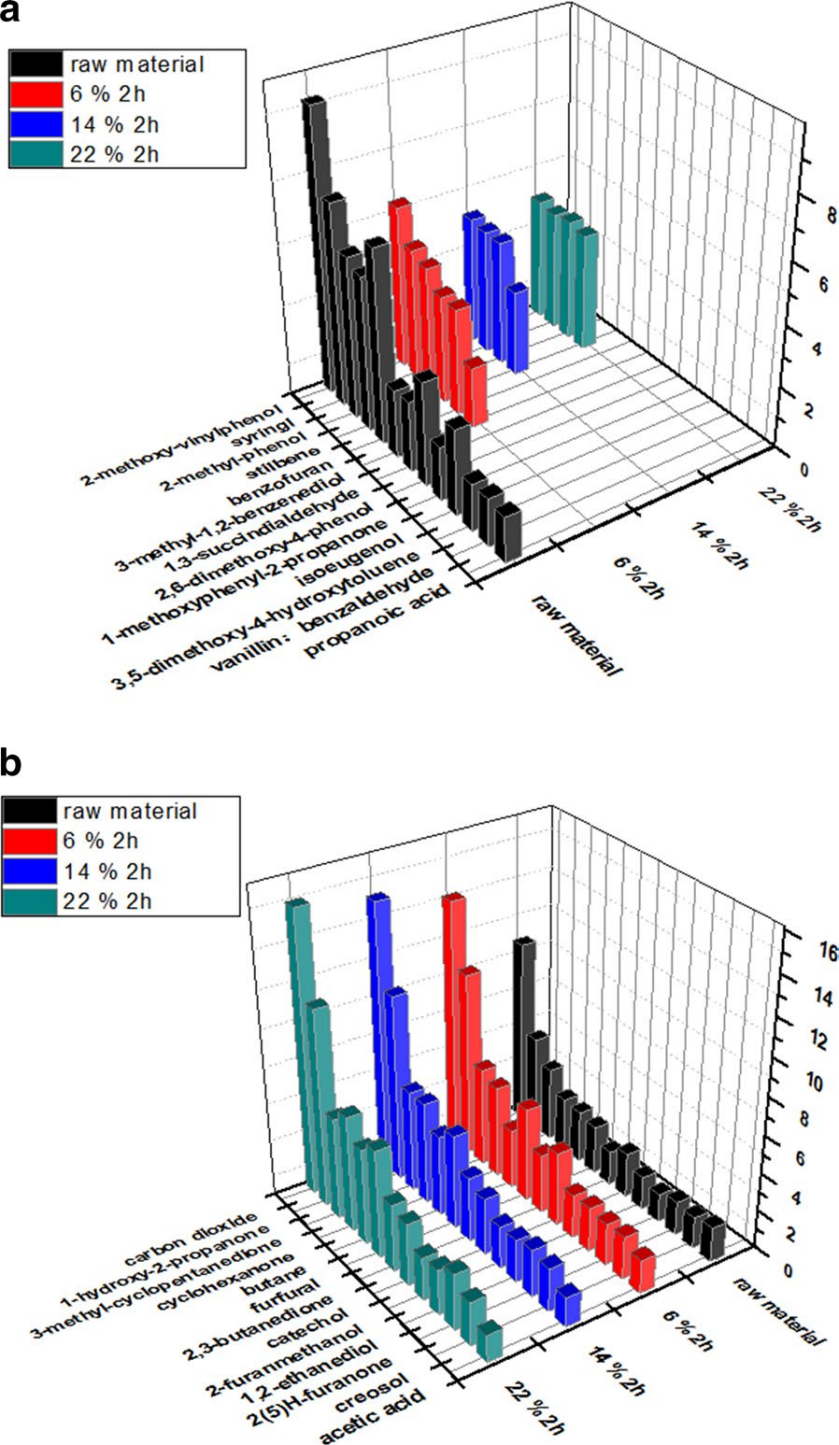
**a** Effect of low-temperature soda extraction on the lignin-derived pyrolysis products identified from GC/MS. **b** Effect of low-temperature soda extraction on the polysaccharide-derived pyrolysis products identified from GC/MS

Lignin-derived pyrolysis products are displayed in Fig. 3a. Since lignin was removed after alkali extraction, the content of pyrolysis products largely decreased. Even so, some kinds of phenolic products such as benzenediol, 2,6-dimethoxyphenol, and isoeugenol disappeared. Those products that disappeared comprised 20% of total production. This indicates that after extraction fewer species would occupy a greater proportion. That is good news for the enrichment in high-value bio-conversion products.

Polysaccharide-derived products are displayed in Fig. 3b. As shown in the “TG–FTIR analysis” section, CO_2_ is one of the important products, and it can be as much as 12.5% of the entire fast pyrolysis production in unextracted straw. After alkali extraction, the content of CO_2_ increases to 15%. CO_2_ is presumably produced in primary reactions or the early stage of cellulose pyrolysis [20]. The other main products are ketones such as acetone, butanedione, cyclohexanone, and furanone, which account for approximately 12% of unextracted straw. After pretreatment, these kinds of products would reach 25%. The proportion of ketone is facilitated by the cleavage of the 1,4-glycosidic linkage in the cellulose polymer, followed by intramolecular rearrangement of the monomer units [21].

However, overloading the NaOH pretreatment could not bring about a higher yield of furanic compounds. When the NaOH loading reaches 22%, an increase of only 0.05% could be gained compared with the 14% NaOH loading. For the polysaccharide-derived products, the contents of most of these products slightly increased.

Amongst extracted straw with different NaOH loadings, it is worth noting that extracted straw using a high NaOH loading (above 14%) is clearly selective for pyrolysis products. On one hand, many lignin pyrolysis products disappeared, and only four kinds of lignin monomer products remained. These pyrolysis products were mainly released in two time periods centred on 19–21 and 40–50 min. On the other hand, the polysaccharide-derived products were enriched. The expected CO_2_, propanone, and furfural peaks have outstanding intensity which could include approximately 30% of the total pyrolysis products. That was good news for separation and the application of pyrolysis to get high-value products.

## Discussion

### Effect of soda extraction on the elemental composition and pore structure

The soda extraction of lignocellulosic materials is a process in which the alkaline lignin, carbohydrate, ash, and alkali-extractable materials are dissolved out. These results reflected that a high NaOH loading has a slight degradation effect on cellulose. Although alkali extraction at such a low temperature (90 °C) was not enough to cause any prominent change in the crystal region, some glycosidic bonds of cellulose can be broken during alkali pretreatment [22]. Lignin and pentosan in the straw were removed by more than 90 and 60%, respectively, when the NaOH loading reached 22%.

Pentosan and lignin largely dissolving out of the straw could result in the distortion of the interior structure and even its collapse. An increase in the NaOH loading can lead to fibre bending and curling, resulting in an increase in pore size increase. This increase in pore volume is beneficial to cracking during pyrolysis, improving the production of volatile matter. However, when the NaOH loading reaches 22%, the collapse of fibre could bring about a decrease in both the pore size and volume. Thus, an appropriate amount of lignin and pentosan retained in the straw could maintain the original interior structure after soda extraction [23].

During the soda extraction process, some part of the surface fibres is pulled out of the surface, resulting in fibrillation of the surface. The BET specific surface area is related to the inner surface area of the pores of the cell wall. The outer fibres of the primary wall and secondary wall are curled after soda extraction. This leads to some amount of the alkali-soluble material penetrating the amorphous regions of the fibre, resulting in fibre swelling. After fibre swelling, the straw cohesion decreases and the inner organisational structure loosens, which results in an increase in the BET specific surface area. However, because of the hydrogen-bonding interaction that occurred when the treated straw was air-dried, the loosened tissue collapsed, shrank, and adjacent fibrous bands closed up, and thus, the excessive addition of lye could bring about structural collapse. Unlike pretreatment with moderate NaOH loading (below 14%), after pretreatment with highly concentrated NaOH solution, the straw could not keep its original structure, which has been shown in our previous work [17].

With alkali-soluble mass dissolving out of the wheat straw, the extracted straw has a high proportion of compounds containing the elements C and H. Obviously, increasing the content of C and H is good for improving the heating value (HV). More than 40% of the mass of the wheat straw could be dissolved out using alkali [24] and which dissolve out first during hot caustic pre-extraction. Because of the higher polarity of oxygen, more oxygenated groups could be contained in the alkali-dissolved mass. Thus, the decrease in oxygen-containing species in the residual straw easily can be understood.

The decrease in the N and S content of the extracted straw can well avoid the release of secondary pollution during the pyrolysis process. During pyrolysis, species containing N and S could be transformed into NO_*x*_ and SO_*x*_ precursors, which are harmful to ambient air quality [25].

### Effect of soda extraction on pyrolysis behaviour and its products

After alkali extraction, fibres could be sufficiently swelled after an extended time of pretreatment. The deformed, bent and even curled fibre is favoured for pyrolysis. In addition, the pyrolysis temperature of carbohydrate is lower than that of lignin. Thus, greater carbohydrate content in the extracted straw could bring about a higher weight loss rate. This indicated that lignin removal could facilitate the pyrolysis of wheat straw and improve the release of volatile components. Hence, extracted straw could lose more weight with a higher weight loss rate than that of unextracted straw.

However, unextracted straw and excessively alkali-extracted straw have a lower pyrolysis temperature. This may be the result of more LMW mass contained in the unextracted straw. This kind of LMW mass has a lower pyrolysis temperature. For excessively alkali-extracted straw, a greater collapse of the interior structure and pores could hinder the pyrolysis process and it can be carbonised to a carbon mass. In addition, previous research [26] also suggested that residual inorganic salts can catalyse the dehydration by scission of both exocyclic and endocyclic C–O bonds. Catalysis can reduce the pyrolysis temperature. Therefore, both untreated straw and straw extracted with a high concentration of NaOH (22%) have lower pyrolysis temperatures. Meanwhile, those untapped and closed fibrous paths could block the volatilisation of pyrolysis gas and more carbide residues could be formed.

Such a phenomenon may result from two reasons. On one hand, volatile organic species were mainly generated by the cracking and reforming of carbohydrate and lignin. Initial thermal decomposition of cellulose is the depolymerisation of the cellulose polymer to form various anhydrosugar derivatives, amongst which levoglucosan is the most prevalent [27]. The formation of levoglucosan from cellulose pyrolysis has been proposed as the cleavage of the 1,4-glycosidic linkage in the cellulose polymer followed by intramolecular rearrangement of the monomer units [28]. After low-temperature alkali extraction, a partial disruption of the 1,4-glycosidic linkage glycosidic linkages [29] had occurred. Therefore, energy for the cracking of the linkages of cellulose polymers could be decreased in the pyrolysis process and more energy could be applied to the cracking and reforming of cellulose.

On the other hand, the structure of the straw plays an important role in pyrolysis behaviour. When the NaOH loading was low, the venation of the vascular bundles of the stem became distinctly undulated after pretreatment and was similar to those of the original straw. Thereafter, pretreatment with 14% NaOH loading not only caused the compact fibrous bands to disperse but also made the surface rough. This was largely the result of the removal of a considerable amount (25%) of soluble components from the straw. This kind of loose structure could offer more paths for pyrolysis gases to be released. Meanwhile, a more-exposed structure produces more reaction area, which is good for cracking lignin and carbohydrate during pyrolysis. Structural units of lignin and carbohydrate could easily be split out through these opened paths. However, when the NaOH loading reached 22%, a large amount (above 45%) of soluble components dissolved out of the extracted straw, and the resulting loosened tissue collapsed and shrank. Adjacent fibrous bands closed up because of the hydrogen-bonding interaction that occurred when the treated straw was air-dried. There was a clear decrease in the pores of the straw after extracting with a 22% NaOH loading. In addition, more release paths for gas products were closed because of structural changes. Therefore, more structural units should be released during the second pyrolysis stage, one step closer to cracking in the third pyrolysis stage (350–600 °C). This may result in the increasing release of CO_2_ whilst more carbonisation was retained in the residues.

Based on the above discussion, after extraction with 14% NaOH loading, considerable soluble mass dissolved out and more pore paths opened whilst the original rigid construction was maintained in the extracted straw. Under this pretreatment condition, the extracted straw could selectively produce monomer units of lignin and carbohydrate during the pyrolysis process. Analysis of the TG–FTIR spectra could clearly demonstrate the trend of weight loss with time and evaluate the functional groups of the volatile matter produced by pyrolysis at a slow heating rate. Py–GC/MS supports ultrarapid heating of samples and can rapidly and accurately distinguish the components of the pyrolysis products. Therefore, the Py–GC/MS technologies can further improve biomass pyrolysis studies at a fast heating rate.

Hot caustic pre-extraction could break the linkages of carbohydrates and achieve their partial hydrolysis. The destruction of these linkages could improve the production of CO_2_ and ketones during fast pyrolysis. The furanic compounds are regarded as a typical ring-containing product from hemicellulose and cellulose. There are two main steps in the production of furfural and HMF; a hydrolysis step that breaks down cellulose and hemicellulose polymers into their six- and five-carbon sugar monomers, and a dehydration step at which the sugars are converted into furanic compounds [30]. After extraction, because of partial decomposition of cellulose and hemicellulose, more sugar monomers could be produced. As the result, more furfural and 2-furfuryl alcohol is released from pyrolysis process.

Based on the above discussion, since lignin is removed from the extracted straw, lignin-derived products declined significantly, especially for phenol products and even some kinds of benzene-series products disappeared. Most of the polysaccharide-derived products increased including the content of many high-value products, such as ketones, increased greatly whilst the content of acids decreased. Both main derived products after soda extraction could facilitate the enrichment of high-quality components.

## Conclusion

Hot caustic pre-extraction can represent a winning and economical strategy to increase pyrolysis properties. After suitable extraction (14% NaOH loading), an appropriate amount of lignin and pentosan retained in the straw could maintain the original interior structure. The loose and exposed structure not only offers greater reaction area for pyrolysis cracking but also provides more paths for the release of pyrolysis products. The interior structure of wheat straw after highly concentrated soda extraction could collapse. The closed paths could hinder the pyrolysis process, and wheat straw can be carbonised to a carbon mass. Therefore, a suitable caustic pre-extraction is needed for efficient pyrolysis of wheat straw into some value-added bio-gas products.

## Methods

### Materials

Wheat straw was harvested from Jining, Shandong province of China in the summer of 2014. It was cut into lengths of 3–5 cm and air-dried for 2 days or more.

### Hot caustic pre-extraction

Hot caustic pre-extraction of the stored straw (100 g) was performed in a 1-L glass beaker and heated in a water bath at 90 °C. NaOH loadings varied from 6 to 22% (g/g dry straw) and a 1:5 solid-to-liquor ratio. The wheat straw was mixed well with the alkali liquor and then poured into the glass beaker. At the end of each pretreatment, the beakers were removed from the water bath and cooled rapidly in cold water. The residues were thoroughly washed with 90 °C distilled water until the wash water was clean. The washed straw was air-dried and then ground and sieved to give a particle size of 40–60 mesh.

### Component analysis of the extracted straw

The analysis of typical composition, including cellulose (Kurschner–Hoffner method), ash (TAPPI 211 om-12), lignin (TAPPI T 222 om-88), acid-insoluble lignin (TAPPI 222 om-11), and pentosan (TAPPI T 223 cm-01), was carried out according to these referenced standard methods. The analyses were conducted in triplicate, and the relative standard deviation was below 5%.

### Elemental analysis

The analysis of the straw elements was performed on a Vario elemental analyser (Germany, Vario company). The measurement parameters were an oxidation furnace temperature of 1150 °C, reduction furnace temperature of 850 °C, carrier gas flow rate of the measuring cell of 90 mL/min, carrier gas flow rate of the reference cell of 20 mL/min, and an oxygen flow rate of 30–80 mL/min.

### Low-temperature nitrogen absorption

Prior to testing, the samples were freeze-dried in order to remove free water. This procedure aids in preserving the pore structure of the fibre [31]. In this study, a pore-size-distribution detector NOVA 4200e (USA, Quantachrome) was used for structural analyses of the fibre pores. High-purity N_2_ was used as the adsorbate and the adsorption–desorption of high-purity N_2_ was determined at 77 K in a liquid nitrogen trap using a static volumetric method. This approach was applied to obtain adsorption–desorption isotherms for the extracted and unextracted straw. Subsequently, calculations based on the BET equation (Brunauer–Emmett–Teller), using the BJH mode, H–K mode, DFT (density functional theory), T-plot, etc., were used to analyse the specific surface area of the porous materials, the sizes of the macro-, meso-, and micro-pores, the total pore volume, average pore size, and surface structural parameters.

### TG–FTIR analysis

The thermogravimetric (TG) analyser (STA449 F3 Jupiter, NETESCH) was coupled with an FTIR spectrometer (TENSOR 27, BRUKER) to determine the mass loss of the straw and online evolution of gaseous products. The experiments were done on the TGA at a heating rate of 20 °C/min within the temperature range 25–800 °C. High-purity nitrogen was used as the carrier gas with a flow rate of 20 mL/min. Approximately 30 mg of sample was placed in the ceramic crucible each time. The FTIR spectrum was obtained in the range 4000–400/cm.

### Py–GC/MS analysis

A pyrolysis–gas chromatography/mass spectrometry (Py–GC/MS) system was used to separate and identify the pyrolysis products. This system is composed of a pyrolyser (CDS 5200, Chemical Data Systems, USA) connected to an Agilent 7890A gas chromatograph (GC) mated to an Agilent 5975C mass spectrometer (MS). The valve oven and transfer lines were maintained at 300 and 285 °C, respectively. The test parameters for the pyrolyser operation are as follows: sample mass, 0.5 mg; carrier gas, N_2_ (99.999%) with a flow rate of 1 mL/min; pyrolysis temperature, 500 °C; heating rate, 20 °C/s; holding time, 30 s. Separation of volatile pyrolysis products was achieved on an Agilent DB-17 ms capillary column (30 m × 0.25 mm × 0.25 μm film thickness). The split ratio was 50:1 with a helium carrier gas flow of 1 mL/min. The GC oven temperature was held at 40 °C for 4 min, programmed to 230 °C (2 min) at 5 C/min, and then heated further to 280 °C (5 min) at 10 °C/min. The parameters for GC/MS operation are as follows: injector temperature, 200 °C; GC/MS interface temperature, 300 °C; mass spectrometer, EI mode at 70 eV; mass spectra, from *m*/*z* 20 to 400 with a scan rate of 500 amu/s. Peak identification was carried out according to the NIST MS library and relevant literature (Additional file 1).

## Additional file

**Additional file 1: Table S1.** Identification Pyrolysis products of wheat straw. **Figure S1.** Py–GC/MS pyrogram of unextracted wheat straw. **Figure S2.** Py–GC/MS pyrogram of unextracted wheat straw after 6% NaOH loading extracted. **Figure S3.** Py–GC/MS pyrogram of unextracted wheat straw after 14% NaOH loading extracted. **Figure S4.** Py–GC/MS pyrogram of unextracted wheat straw after 22% NaOH loading extracted.
