# Supplementary material for: Reinforcement of the bio-gas conversion from pyrolysis of wheat straw by hot caustic pre-extraction
Source: Biotechnol Biofuels. 2018 Mar 19;11:72. doi: 10.1186/s13068-018-1072-5 (PMC5858128; doi:10.1186/s13068-018-1072-5)
Supplement: Supplementary file 1 — Additional file 1: Table S1. Identification Pyrolysis products of wheat straw. Figure S1. Py–GC/MS pyrogram of unextracted wheat straw. Figure S2. Py–GC/MS pyrogram of unextracted wheat straw after 6% NaOH loading extracted. Figure S3. Py–GC/MS pyrogram of unextracted wheat straw after 14% NaOH loading extracted. Figure S4. Py–GC/MS pyrogram of unextracted wheat straw after 22% NaOH loading extracted. [file 13068_2018_1072_MOESM1_ESM.doc]

**Additional information**

**Table S1. Identification Pyrolysis products of wheat straw**

| **Peak** | **Retention time (min)** | **Compound** | **Formula** |
| --- | --- | --- | --- |
| 1 | 1.589 | [carbon](../../../../D:/Program%20Files/Youdao/Dict/6.3.69.8341/resultui/frame/javascript:void(0)%3B) [dioxide](../../../../D:/Program%20Files/Youdao/Dict/6.3.69.8341/resultui/frame/javascript:void(0)%3B) | CO_2_ |
| 2 | 1.924 | 2,3-butanedione | C_4_H_6_O_2_ |
| 3 | 2.708 | 2-propanone,1-hydroxy | C_3_H_8_O_2_ |
| 4 | 3.999 | acetic acid | CH_3_COOH |
| 6 | 4.338 | propanoic acid | [C_3_H_6_O_3_](http://www.ichemistry.cn/cas/C3H6O3) |
| 7 | 4.953 | furfural | C_5_H_4_O_2_ |
| 8 | 5.445 | 2-furanmethanol | C_5_H_6_O_2_ |
| 9 | 5.626 | 1,2-ethanediol | C_2_H_6_O_2_ |
| 10 | 6.432 | 2(5)H-furanone | C_6_H_8_O_3_ |
| 11 | 6.727 | cyclohexanone | C_6_H_10_O |
| 12 | 8.477 | 3-methyl-cyclopentanedione | C_6_H_11_O |
| 13 | 9.386 | Guaiacol (G) | C_7_H_8_O_2_ |
| 14 | 10.975 | creosol | C_8_H_10_O_2_ |
| 15 | 11.284 | catechol | C_6_H_6_O_2_ |
| 16 | 11.446 | benzofuran | C_8_H_6_O |
| 17 | 12.114 | 1,2-benzenediol-3-methyl | C_7_H_8_O_3_ |
| 18 | 12.764 | 2-methoxy-vinylphenol | C_8_H_8_O |
| 19 | 13.267 | 2,6-dimethoxyphenol | C_8_H_10_O_3_ |
| 20 | 13.977 | vanillin；benzaldehyde | C_7_H_6_O |
| 22 | 14.563 | isoeugenol | C_10_H_12_O_2_ |
| 23 | 15.576 | 1-methoxyphenyl-2-propanone | C_10_H_12_O_2_ |
| 24 | 15.963 | stilbene | C_14_H_12_ |
| 25 | 17.55 | 2,6-dimethoxy-4-phenol | C_8_H_10_O_3_ |
| 26 | 20.57 | anthraquinone | C_14_H_8_O_2_ |


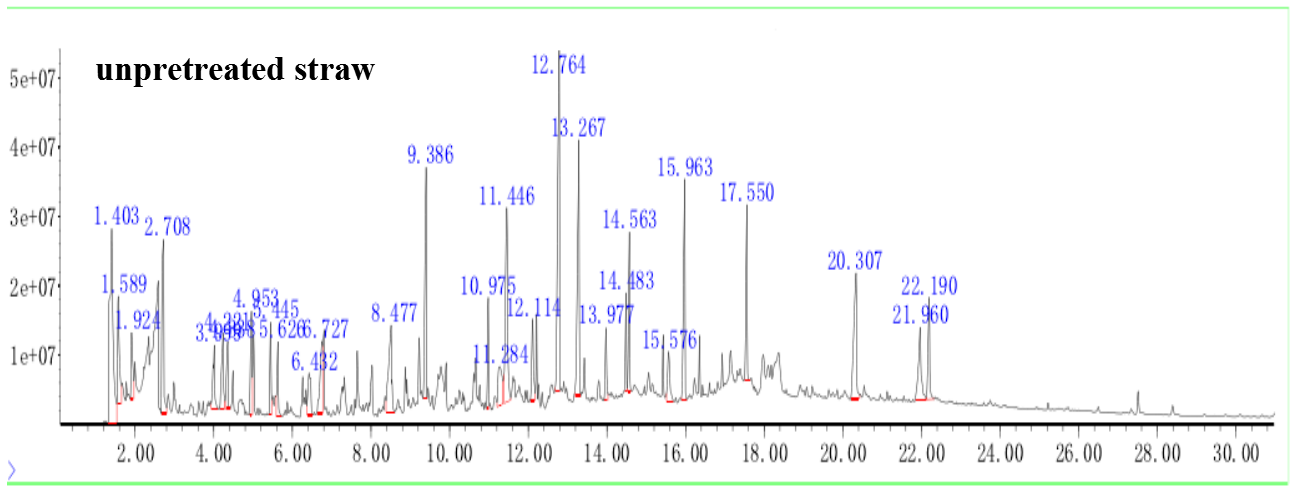


**Figure S1**. Py-GC/MS pyrogram of unextracted wheat straw


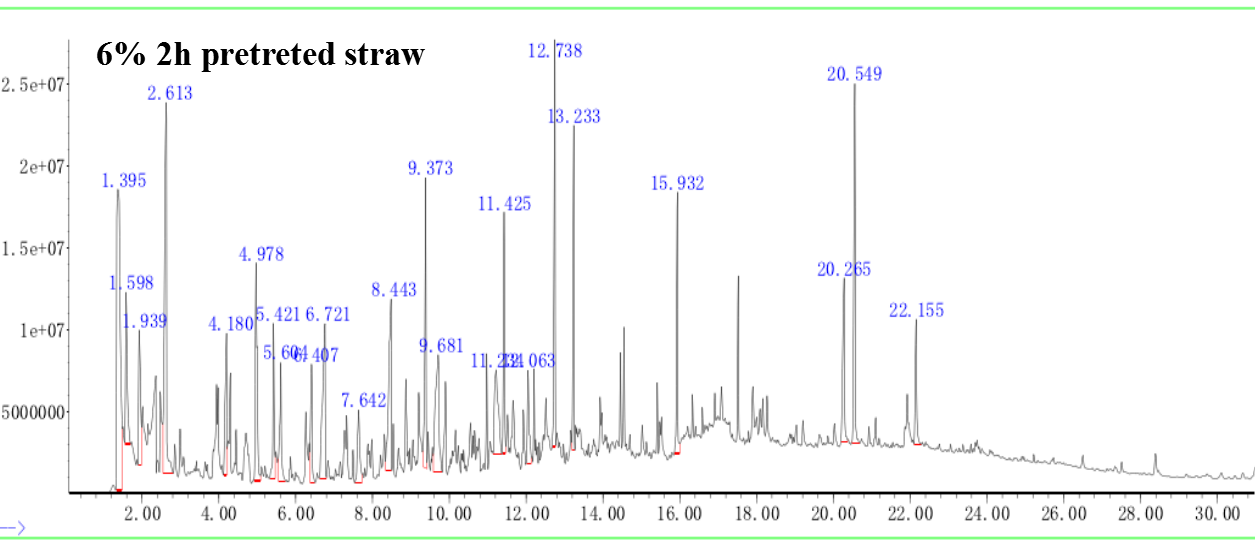


**Figure S2**. Py-GC/MS pyrogram of unextracted wheat straw after 6% NaOH loading extracted


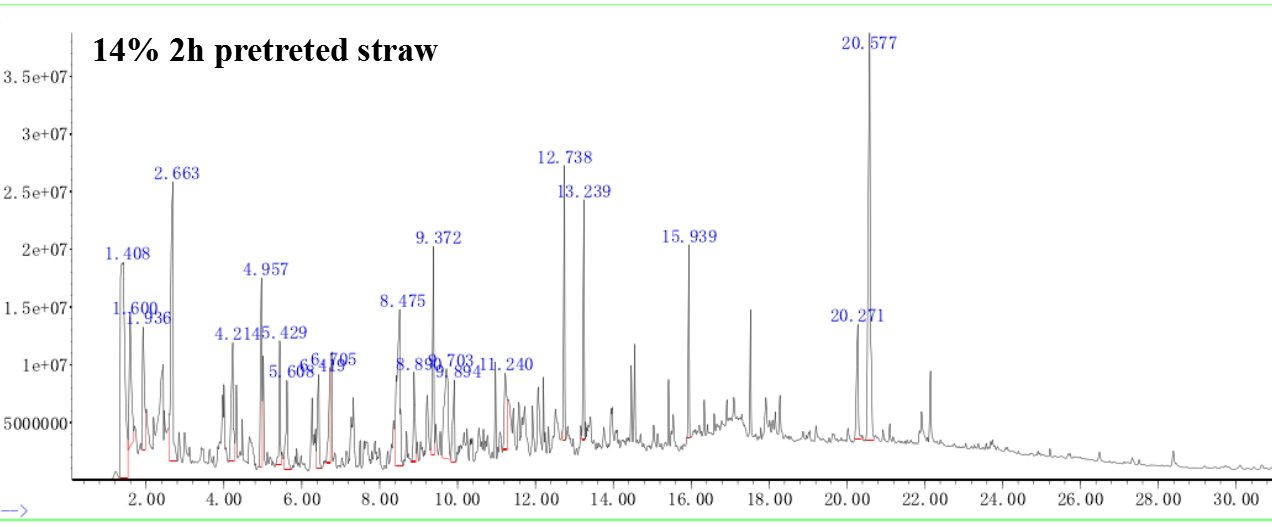


**Figure S3.** Py-GC/MS pyrogram of unextracted wheat straw after 14% NaOH loading extracted


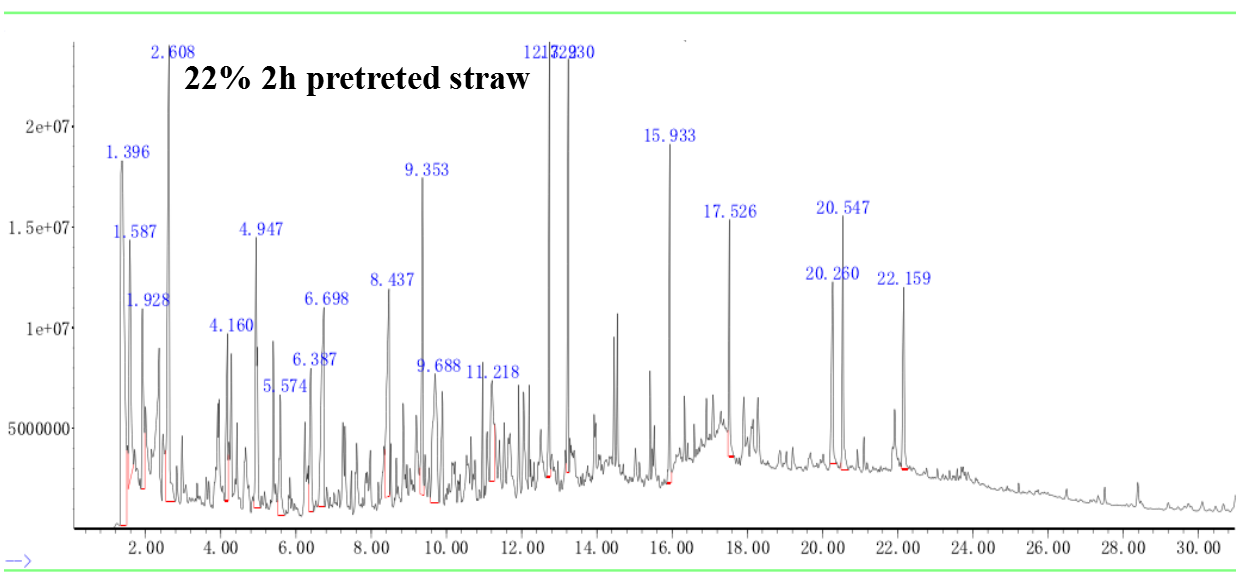


**Figure S4.** Py-GC/MS pyrogram of unextracted wheat straw after 22% NaOH loading extracted
